# Prevalence of Potentially Zoonotic Endoparasites in Domestic Dog Puppies

**DOI:** 10.3390/vetsci12040332

**Published:** 2025-04-03

**Authors:** Gisele Moraes dos Santos Reginaldo, Giovanni Widmer, Sandra Valéria Inácio, Jancarlo Ferreira Gomes, Walter Bertequini Nagata, Gabriela Pinheiro Tirado Moreno, João Alfredo Biagi Camargo Neto, Wagner Luis Ferreira, Felipe Augusto Soares, Katia Denise Saraiva Bresciani

**Affiliations:** 1 Faculdade de Ciências Agrárias e Veterinárias, Universidade Estadual Paulista (Unesp), Jaboticabal 13083-887, Brazil; gisele.ms.reginaldo@unesp.br; 2 Department of Infectious Diseases and Global Health, Cummings School of Veterinary Medicine, Tufts University, Medford, MA 01536, USA; giovanni.widmer@tufts.edu; 3 Faculdade de Medicina Veterinária de Araçatuba, Universidade Estadual Paulista (Unesp), 793 Clóvis Pestana Street, Araçatuba 16050-680, Brazil; sandra_byol@yahoo.com.br (S.V.I.); walter.bn@hotmail.com (W.B.N.); joao.alfredo@unesp.br (J.A.B.C.N.); wagner.luis@unesp.br (W.L.F.); 4 School of Medical Sciences, University of Campinas, Campinas 13083-887, Brazil; jgomes@ic.unicamp.br (J.F.G.); biofefcm@unicamp.br (F.A.S.); 5 School of Veterinary Medicine and Zootechnics, São Paulo State University, São Paulo 05508-220, Brazil; gabipinheirot@hotmail.com

**Keywords:** helminths, protozoa, One Health, dogs, diagnosis

## Abstract

Despite the existence of therapeutic and prophylactic measures, gastrointestinal parasites are common in pets. Due to the zoonotic potential of some species, parasitic protozoa and helminths are of great importance to public health. In this study, we investigated the occurrence of the main gastrointestinal parasites in domestic dog puppies in the city of Araçatuba, São Paulo, Brazil. One hundred fecal samples were collected from dogs up to six months of age. Parasites were diagnosed using Willis’, Faust’s and malachite green coproparasitological techniques. Parasite prevalence as determined by Willis and/or Faust diagnostic techniques was as follows: *Toxocara* spp. 34%, *Cystoisospora* spp. 28%, Ancylostomatidae 22% and *Giardia* spp. 8%. These prevalence rates were calculated by considering an animal to be positive if Willis’ or Faust’s or both tests returned a positive result. *Cryptosporidium* diagnosis with malachite green was negative for all samples. Infection with *Toxocara* spp., the most prevalent pathogen in this survey, was not limited to dogs with abnormal fecal consistency. The occurrence of asymptomatic parasitized dogs increases the risk of zoonotic transmission.

## Simple Summary

Gastrointestinal parasites are common in domestic dogs around the world. Many of these parasites are potentially zoonotic and are important pathogenic agents in public health. This is the first study of the occurrence of gastrointestinal parasites in domesticated puppies under six months of age. Samples were collected from 100 randomized animals of both sexes and occurrence of *Toxocara* spp., *Cystoisospora* spp., the Ancylostomatidae family and *Giardia* spp. was found. Toxocariasis in asymptomatic dogs highlights the risk of zoonotic transmission.

## 1. Introduction

Some gastrointestinal parasites are of interest from a One Health perspective because of their veterinary and public health relevance. Visceral Larva Migrans and Cutaneous Larva Migrans caused by *Toxocara* spp. and the Ancylostomatidae family, respectively, are examples of such parasites as well as Giardiasis which is caused by *Giardia* spp. assemblages [1,2,3,4]. Helminth eggs and protozoan cysts excreted in the environment by domestic animals should be a matter of concern to their owners due to the importance that animals can have for emotional support and their role in social and physical development of people, particularly children and the elderly [5,6,7,8]. Close co-habitation of humans and domestic animals, which are sometimes view as members of the family, favors zoonotic transmission [9,10,11]. In this context, it is particularly important to investigate the occurrence of parasites in domiciled dogs.

Various parasite species are diagnosed with moderate to high frequency in companion animals despite the existence of therapeutic and prophylactic measures [8,12]. Diarrhea resulting from parasitic infections and from other causes is one of the most frequent disorders in canine puppies and negatively interferes with their growth [8,13]. Thus, it is essential to prevent or minimize parasite transmission. Screening for the presence of gastrointestinal parasites [2,14] informs treatment options and the adoption of environmental management practices for infection control [10,13,15]. In contrast with surveys, which include a wide age range, we investigated the occurrence of gastrointestinal parasites exclusively in domiciled dogs up to six months of age in Araçatuba, Brazil.

## 2. Materials and Methods

### 2.1. Study Population

The minimum sampling required for the execution of this project, at the 95% confidence level and with absolute precision of 10%, was determined to be 96 samples, based on a prevalence of 50% [16]. Thus, we collected fecal samples from 100 randomly selected dogs, 60 males and 40 females, that were all domiciled and of mixed breed, all aged less than six months and all from Araçatuba region, Brazil. Within this age range, 20 animals were one to two months old, 44 animals were three to four months old and 36 animals were five to six months old. Puppies aged between 2 and 3 months and between 4 and 5 months were not represented in the sample. This study was approved by the animal use ethics committee of the Faculty of Dentistry of Araçatuba, São Paulo State University, under protocol number FOA-00312-2016.

### 2.2. Degree of Dehydration and Fecal Consistency

The degree of dehydration was assessed as not apparent, mild, moderate, severe and shock [17]. Fecal consistency was assessed visually and defined as liquefied, pasty, semi-solid and solid, according to Coelho et al., 2012 [18].

### 2.3. Collection of Fecal Material and Parasite Detection

With the aid of a urethral catheter (n° 6-8-10) and a 10 mL syringe, feces were collected directly from the rectal ampulla. Samples were stored in sterile vials and refrigerated between 4 °C and 8 °C until processed. Subsequently, Faust’s flotation technique in saturated sodium chloride solution [19] and Willis’ centrifuge–flotation technique in zinc sulfate [20] were applied. The presence of parasites eggs and cysts was also examined by negative malachite green staining [21]. All samples were examined with the three diagnostic techniques.

## 3. Results

Based on at least one positive test, 34% of dogs were positive for *Toxocara* spp., 28% for *Cystoisospora* spp., 22% for the Ancylostomatidae family and 8% for *Giardia* spp. In 21% of the animals, mixed infections were detected. One or more parasite species were detected in 62% of dogs with at least one technique. Among the 100 dogs evaluated in the study, with the Willis technique we detected 32% animals positive for *Toxocara* spp., 19% the Ancylostomatidae family and 16% for *Cystoisospora* spp., including 16% that presented mixed infections with the Ancylostomatidae family, *Cystoisospora* spp. and *Toxocara* spp. Faust’s technique identified 30% of dogs with *Toxocara* spp., 26% with *Cystoisospora* spp., 10% with the Ancylostomatidae family and 8% with *Giardia* spp. Based on this technique, 21% of the animals were infected with more than one parasite. For 82% of the samples, Faust and Willis gave concordant results in the sense that both tests were negative or both were positive. Of the 18 (18%) discordant results, 12 were positive according to Willis and negative according to Faust. For six samples, the results were reversed (chi-square = 39.0, *p* < 0.001). Six of six Willis-negative/Faust-positive samples were Faust-positive for *Cystoisospora* spp., indicating that the Willis technique may underestimate the prevalence of this parasite. Conversely, of the 12 samples positive by Willis and negative by Faust, Ancylostomatidae was detected by itself or in combination with a second parasite in 10 samples. Of the 44 double-positive samples, 12 were diagnosed with different parasites or a different combination of parasites where more than one pathogen was detected. The diagnosis for the remaining 32 samples was fully concordant.

Dehydration was not apparent in 50 dogs while the same number of dogs were found to be mildly dehydrated. Regarding the consistency of fecal samples, 43 samples were scored as liquefied, 7 as pasty, 8 as semi-solid and 42 as solid. Dogs excreting liquefied and solid feces presented more positive than dogs with pasty and semi-solid feces. In particular, it is important to note that 40 of 53 puppies excreting solid feces were positive for gastrointestinal parasites (Table 1). There was no significant association between fecal consistency and presence of parasite (Fisher’s exact test, *p* = 0.8589).

No association between age and parasite prevalence was observed using linear regression (r = 0.05, *p* = 0.56). Similarly, sex and infection were not significantly associated. A total of 65% of males and 57.5% of females were positive by at least one diagnostic technique (chi-square = 0.30, *p* = 0.58). The percentage prevalence for the oldest age group is 29.4% for *Toxocara*, 45.5% for Ancylostomatidae, and 35.7% for *Cystoisospora*. However, the number of positive animals at age 5-6 months is 10 for each of the 3 parasites (Table 2). An opposite trend was observed for Ancylostomatidae which increased in prevalence with age from 5% to 45%.

## 4. Discussion

Our results extend the epidemiological study of common gastrointestinal helminths and protozoa of dogs to puppies up to six months of age. Typically, coproparasitological surveys include animals of any age [2,14,22,23]. Other studies have reported the occurrence of gastrointestinal parasites, but they differ from this study since they frequently examine stray dogs regardless of age [15,24,25,26,27,28]. Therefore, a limitation observed in our study is the lack of information about the presence of older dogs sharing the same spaces of the puppies investigated.

The main finding of our survey is the frequent detection of *Cystoisospora* spp., *Giardia* spp., *Toxocara* spp. and the Ancylostomatidae family in puppies with varied fecal consistencies, including animals showing no symptoms typically associated with intestinal parasites. This observation is relevant to public health as the latter three species are zoonotic [3,4,25,26,27,28,29]. As puppies are more likely to excrete gastrointestinal parasites [24,29,30,31], deworming and other measures to reduce transmission are particularly important to reduce the risk of infection, which can have severe consequences in immunocompromised children and adults. Such measures are also expected to benefit the health of puppies [24,32]. Mixed infections with two, or even three, parasites were relatively common in our survey. This observation emphasizes the need for adequate medications to treat helminth and protozoan co-infections.

The use of two flotation techniques supports the conclusion that both methods have similar sensitivity. This observation is consistent with the fact that both methods concentrate parasite eggs and cysts by flotation in a high-density salt solution. The difference in *Toxocara* spp. and *Cystoisospora* spp. prevalence based on Faust and Willis raises interesting questions about the buoyant properties of these eggs and cysts and may justify the use of both methods where the presence of these parasites is suspected [2,14,33]. This recommendation should be easy to implement as fecal flotation is cheap and easy to perform [14]. Veterinarians should make owners aware of the importance of diagnosing these parasites, particularly given the high prevalence of *Toxocara* spp. and its potential for zoonotic transmission [11,34,35,36].

Malachite green staining did not reveal the presence of *Cryptosporidium* spp. oocysts. This stain has the advantage of being cheaper and easier to perform than immunological and molecular assays [37], but its disadvantage is low sensitivity, with the possibility of false negative results [38]. The oocysts have small dimensions and as such are hard to observe in fecal smears after staining, which requires more time and observational skill from an examiner [39].

## 5. Conclusions

We investigated, for the first time in Brazil, the occurrence of gastrointestinal parasites in domestic puppies less than six months of age. The detection of intestinal helminths as *Toxocara* spp. and the Ancylostomatidae family and the protozoa *Giardia* spp. in asymptomatic dogs highlights the risk of zoonotic transmission.

## Figures and Tables

**Table 1:** Number of domestic dogs positive for gastrointestinal parasites by fecal consistency.

Parasite	Consistency of Fecal Samples				
	Liquefied	Pasty	Semi-Solid	Solid	Total
*Toxocara* spp.	14	3	5	15	37
Ancylostomatidae	8	1	2	11	22
*Giardia* spp.	5	0	0	3	8
*Cystoisospora* spp.	12	0	5	11	28
No parasites	19	3	3	13	38

**Table 2:** Number of positive domestic dogs for gastrointestinal parasites according to age *.

Age Range (Months)	Gastrointestinal Parasites															
	*Toxocara* spp.				Ancylostomatidae				*Giardia* spp.				*Cystoisospora* spp.			
	Positive	%	Negative	%	Positive	%	Negative	%	Positive	%	Negative	%	Positive	%	Negative	%
1–2	9	26.5	11	16.7	1	4.5	19	24.4	2	25	18	19.6	8	28.6	12	16.7
3–4	15	44.1	29	43.9	11	50.0	33	42.3	4	50	40	43.5	10	35.7	34	47.2
5–6	10	29.4	26	39.4	10	45.5	26	33.3	2	25	34	36.9	10	35.7	26	36.1
Total	34	100	66	100	22	100	78	100	8	100	92	100	28	100	72	100

* Positive by one or both flotation diagnostic methods.

## Data Availability

The original contributions presented in this study are included in the article. Further inquiries can be directed to the corresponding author.
